# Fullerenol C_60_(OH)_36_ Protects the Antioxidant Enzymes in Human Erythrocytes against Oxidative Damage Induced by High-Energy Electrons

**DOI:** 10.3390/ijms231810939

**Published:** 2022-09-19

**Authors:** Jacek Grebowski, Paulina Kazmierska-Grebowska, Natalia Cichon, Anna Konarska, Marian Wolszczak, Grzegorz Litwinienko

**Affiliations:** 1 Department of Molecular Biophysics, Faculty of Biology and Environmental Protection, University of Lodz, Pomorska 141/143, 90-236 Lodz, Poland; 2 The Military Medical Training Center, 6-Sierpnia 92, 90-646 Lodz, Poland; 3 Department of Neurobiology, Faculty of Biology and Environmental Protection, University of Lodz, Pomorska 141/143, 90-236 Lodz, Poland; 4 Biohazard Prevention Centre, Faculty of Biology and Environmental Protection, University of Lodz, Pomorska 141/143, 90-236 Lodz, Poland; 5 Institute of Applied Radiation Chemistry, Technical University of Lodz, Wroblewskiego 15, 93-590 Lodz, Poland; 6 Faculty of Chemistry, University of Warsaw, Pasteura 1, 02-093 Warsaw, Poland

**Keywords:** fullerenol, ionizing radiation, radioprotection, erythrocytes, antioxidant enzymes

## Abstract

Ionizing radiation (IR) can pass through the human body easily, potentially causing severe damage to all biocomponents, which is associated with increasing oxidative stress. IR is employed in radiotherapy; however, in order to increase safety, it is necessary to minimize side effects through the use of radioprotectors. Water-soluble derivatives of fullerene exhibit antiradical and antioxidant properties, and these compounds are regarded as potential candidates for radioprotectors. We examined the ability of fullerenol C_60_(OH)_36_ to protect human erythrocytes, including the protection of the erythrocytal antioxidant system against high-energy electrons. Human erythrocytes irradiated with high-energy [6 MeV] electrons were treated with C_60_(OH)_36_ (150 µg/mL), incubated and haemolyzed. The radioprotective properties of fullerenol were determined by examining the antioxidant enzymes activity in the hemolysate, the concentration of -SH groups, as well as by determining erythrocyte microviscosity. The irradiation of erythrocytes (650 and 1300 Gy) reduces the number of thiol groups; however, an attenuation of this harmful effect is observed (*p* < 0.05) in the presence of C_60_(OH)_36_. Although no significant effect of fullerenol was recorded on catalase activity, which was preserved in both control and test samples, a more active protection of other enzymes was evident. An irradiation-induced decrease in the activity of glutathione peroxidase and glutathione reductase became an increase in the activity of those two enzymes in samples irradiated in the presence of C_60_(OH)_36_ (*p* < 0.05 and *p* < 0.05, respectively). The fourth studied enzyme, glutathione transferase, decreased (*p* < 0.05) its activity in the irradiated hemolysate treated with C_60_(OH)_36_, thus, indicating a lower level of ROS in the system. However, the interaction of fullerenol with the active centre of the enzyme cannot be excluded. We also noticed that radiation caused a dose-dependent decrease in the erythrocyte microviscosity, and the presence of C_60_(OH)_36_ reduced this effect (*p* < 0.05). Overall, we point to the radioprotective effect of C_60_(OH)_36_ manifested as the protection of the antioxidant enzymes of human erythrocytes against IR-induced damage, which has not been the subject of intense research so far.

## 1. Introduction

Ionizing radiation (IR) can pass through the human body easily, potentially causing severe damage to all biocomponents, including DNA [1]. The deleterious effects of IR are strongly associated with increasing oxidative stress [2], resulting from the ionization of both organic and inorganic compounds [3], with the radiolysis of water as the main process contributing to the increased formation of reactive oxygen species (ROS) in the cell [3,4]. ROS rapidly react with biomolecules, leading to cell dysfunction and cell death [2]. ROS generated by water radiolysis cause damage of the so-called “antioxidant enzymes” and, as a consequence, cause a general decrease in their activity [5,6,7]. Although the above-mentioned processes are employed in modern radiotherapy targeting cancer cells, the side effects need to be minimized to make radiotherapy as safe as possible. For this reason, it is undoubtedly important to identify new, safe and effective nanocompounds with the ability to protect healthy cells from IR-induced damage. Oxidative stress is frequently described as an imbalance between the production of ROS in biological systems and their ability to defend through the sophisticated antioxidant machinery. Erythrocytes are often chosen as a model for studies on cell homeostasis, being continuously threatened by oxidative events, associated to high ROS levels [8].

Water-soluble fullerene derivatives with hydroxy groups exhibit antioxidant properties [9,10,11,12], and these compounds with general formula C_60_(OH)_n_ and M@C_82_(OH)_n_ are regarded as potential candidates for radioprotectors during IR-incused damage [7,10,12]. There are a handful of studies indicating that fullerenols might perform the role of protective agents due to their ability to effectively scavenge free radicals and reduce the lipid peroxidation of the plasma membrane. They maintain the redox balance in the body by protecting or stimulating the activity of antioxidant enzymes or by mimicking their activity [9,13,14,15,16,17]. Hydrophilic properties and the ability to scavenge free radicals make fullerenols a serious alternative to the currently used pharmacological methods in radiobiology. Interestingly, the antioxidant capability of Gd-containing metallofullerene was evidenced under conditions of model oxidative stress [18]. Moreover, antioxidant and anti-inflammatory properties of non-functionalized fullerenes were confirmed on cellular models [19,20].

The harmful effects of IR on living organisms and cells are mainly mediated by ROS, and the radioprotective effect of the fullerenols of C_60_(OH)_24_ was demonstrated in vivo by studies conducted on healthy mice and rats. Trajković et al. [14] compared the properties of fullerenol C_60_(OH)_24_ (100 mg/kg) with the standard commercial radioprotector amifostine (300 mg/kg). They found that both compounds prolonged the life of rats irradiated with a lethal dose of X-rays. Fullerenol C_60_(OH)_24_ more effectively protected against oxidation in the number of granulocytes and lymphocytes, especially on the seventh and fourteenth day after irradiation. Pathohistological examinations revealed better radioprotective effects of C_60_(OH)_24_ compared to those of amifostine on the spleen, small intestine, and lung. Fullerenol C_60_(OH)_24_ administered to mice within a fortnight at a daily dose of 40 mg/kg had no side effects in non-irradiated mice. It reduced mortality after the whole-body exposure to a lethal dose of X-rays. C_60_(OH)_24_ exhibited a radioprotective effect by enhancing immune system functions, increasing antioxidant enzyme activity, and protecting the mitochondrial membrane, thereby inhibiting the apoptosis process [7]. In vitro studies revealed that *Stylonychia mytilus* cells exposed to ^60^Co gamma-rays were protected by fullerenol in a concentration-dependent manner [15]. The beneficial effects of fullerenol were observed when it was used at a concentration of 0.1 mg/mL, while the irradiation dose was of 100–1500 Gy. Along with the increase in fullerenol concentration and the radiation dose (2000 Gy), a toxic effect was observed due to the intensification of the lipid peroxidation process [15]. The radioprotective effect of C_60_(OH)_24_ was also demonstrated in vitro in human acute myeloid leukaemia cell line K562. No morphological changes in fullerenol-treated cells were observed to confirm the lack of cytotoxic effect, whereas an X-ray dose of 24 Gy resulted in a reduction in viability, a loss of colony-forming ability, changes in morphology, and a decrease in antioxidant enzyme activity. The treatment of cells with C_60_(OH)_24_ caused a partial protection against the aforementioned harmful effects of radiation, and had a positive effect on the cell number, cell morphology, and the activity of antioxidant enzymes [21]. Interestingly, Zhao et al. [22] showed that fullerenols significantly blocked the ROS-induced damage and enhanced the viability of irradiated human keratinocyte cells in vitro. The same authors performed in vivo studies and reported that medical sodium hyaluronate hydrogels loaded with fullerenols were suitable for skin administration and powerfully mitigated radiodermatitis via effectively protecting epidermal stem cells, thus, promoting the fullerenols as potential skin radioprotectors [22]. In another study, a fullerenol containing nanocomposite (FNMT: fullerenol@nano-montmorillonite) was developed and tested in protecting duodenum damage caused by X-ray irradiation in mice. FNMT was found to significantly reduce radiation-induced diarrhoea, weight loss, and duodenum tissue pathological damage [23].

Due to their unique properties, fullerenes and fullerenols may be also used to generate ROS and could be used in radiotherapy as radiosensitizers by an augmentation of the amount of oxidative damage in radiation-treated cancer cells [24,25,26].

The results of the above sparse studies encouraged us to investigate whether fullerenol C_60_(OH)_36_ have the ability to protect human erythrocytes and their antioxidant system against high-energy electrons.

## 2. Results

**Figure 1:**
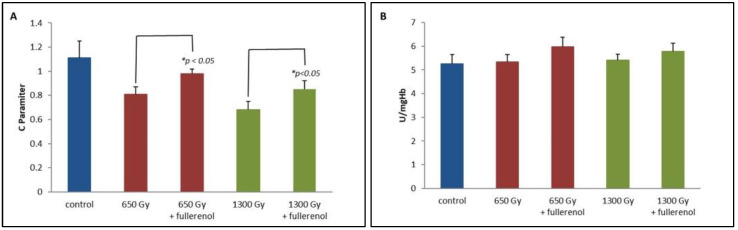
(**A**) The influence of fullerenol on the C parameter determined using the RSSR spin tracer. Fullerenol at a concentration of 150 µg/mL was added to the erythrocyte suspension 1 h before the irradiation with doses of 650 and 1300 Gy. Measurements were performed 2 h after irradiation. The bars represent mean values ± SD for 3 independent measurements. (**B**) The effect of fullerenol on catalase (CAT) activity. Fullerenol at a concentration of 150 µg/mL was added to the erythrocyte suspension 1 h before the irradiation with a dose of 650 or 1300 Gy. Measurements were taken 2 h after the irradiation and haemolysis. The bars represent mean values ± SD for 3 independent measurements. * Statistically significant values at *p* < 0.05.

In this study, the level of oxidative damage to biomolecules was measured by monitoring the concentration of -SH groups of proteins (expressed as the parameter C being a marker of thiol groups, as described in our previous paper [9]). For the samples not treated with fullerenol exposed with 650 or 1300 Gy, the parameter C decreased by 27% (*p* < 0.05) and 38% (*p* < 0.05), respectively (in relation to the control, see Figure 1A), wherein a 650 Gy dose compared to the control caused a decrease in the parameter C by about 12% (*p* < 0.05) for fullerenol-treated samples. Moreover, the radiation dose of 1300 Gy was associated with the depletion of the thiol groups by about 24% (*p* < 0.05) in the C_60_(OH)_36_-group in comparison to the control group (Figure 1A).

The effect of irradiation in the presence of fullerenol on catalase (CAT) activity is presented in Figure 1B. After incubating the Red Blood Cells (RBC) suspension with C_60_(OH)_36_ subjected to irradiation, the erythrocytes were subjected to haemolysis, and CAT activity was measured (see the Materials and Methods for details). As shown on the graph, the presence of C_60_(OH)_36_ conserved and even slightly increased (*p* < 0.05) CAT activity after 2 h of irradiation with doses of 650 or 1300 Gy, in comparison to the samples that were irradiated without a treatment with C_60_(OH)_36_.

**Figure 2:**
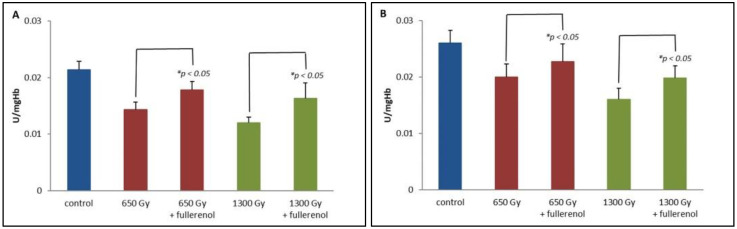
(**A**) The effect of fullerenol on glutathione peroxidase (GPx) activity. Fullerenol at a concentration of 150 µg/mL was added to the erythrocyte suspension 1 h before irradiation with doses of 650 and 1300 Gy. Measurements were taken 2 h after irradiation and haemolysis. Bars represent mean values ± SD for 3 independent measurements. (**B**) The effect of fullerenol on glutathione reductase (GSR) activity. Fullerenol at a concentration of 150 µg/mL was added to the erythrocyte suspension 1 h before irradiation with doses of 650 or 1300 Gy. Measurements were taken 2 h after irradiation and haemolysis. * Statistically significant values at *p* < 0.05.

Figure 2A shows the GPx activity in the RBC suspension incubated with 150 μg/mL of C_60_(OH)_36_, irradiated (650 or 1300 Gy) and subsequently subjected to haemolysis. For samples irradiated without C_60_(OH)_36_, IR caused a decrease in GPx activity after 2 h from irradiation, and these changes are statistically significant with respect to non-irradiated controls (*p* < 0.05). However, the presence of C_60_(OH)_36_ resulted in a significant increase in enzyme activity, although the GPx activity did not reach the level of the control sample.

We also measured the effect of fullerenol for glutathione reductase (GSR) activity. GSR activity in the RBC suspension incubated with 150 μg/mL of C_60_(OH)_36_ and subjected to irradiation of 650 or 1300 Gy is presented in Figure 2B. A decrease in GSR activity after 2 h from irradiation is statistically significant, and the changes are proportional to the dose (with respect to controls for *p* < 0.05). For the irradiated samples containing C_60_(OH)_36_, we observed an increase (at *p* < 0.05) of the reductase activity in respect to irradiated samples not treated with C_60_(OH)_36_.

**Figure 3:**
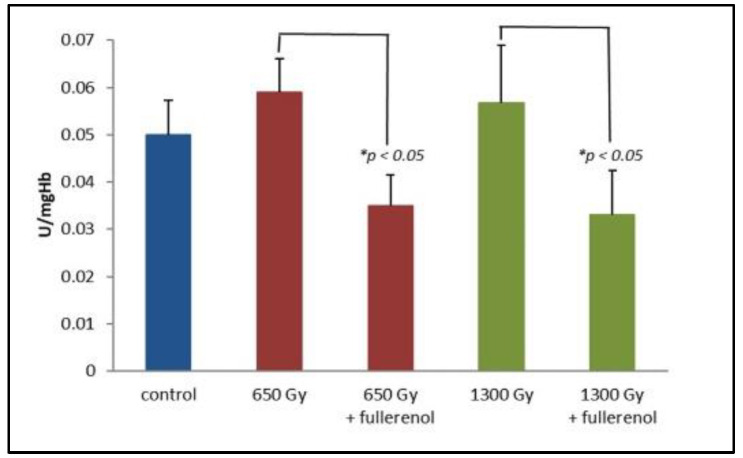
The effect of fullerenol on glutathione transferase (GST) activity. Fullerenol at a concentration of 150 µg/mL was added to the erythrocyte suspension 1 h before irradiation with doses of 650 and 1300 Gy. Measurements were taken 2 after irradiation and haemolysis. The bars represent mean values ± SD for 3 independent measurements. * Statistically significant values at *p* < 0.05.

Figure 3 shows the changes in glutathione transferase (GST) activity. For samples without fullerenol, IR causes a non-significant increase in GST activity after 2 h from irradiation (650 Gy or 1300 Gy) with respect to controls for *p* < 0.05. In contrast, a statistically significant (at *p* < 0.05) decrease in enzymatic activity is observed for samples irradiated in the presence of 150 μg/mL of C_60_(OH)_36_.

The final series of experiments were devoted to the influence of irradiation in the presence of C_60_(OH)_36_ on erythrocyte internal microviscosity. In order to examine such an effect, isolated erythrocytes with a haematocrit of 2% were incubated with 150 μg/mL of C_60_(OH)_36_ for 1 h at 37 °C and then irradiated with doses of 650 or 1300 Gy. Measurements were made using the Tempamine spin tracer, which penetrates relatively easily through the plasma membrane into the erythrocyte interior [27], and the method compares the value of the rotational correlation time of Tempamine inside the cell, referring to a solution with known viscosity [9,28]. The results, expressed as η parameters, are shown in Table 1. It is clear that radiation causes a dose-dependent decrease in the erythrocyte microviscosity and C_60_(OH)_36_ present in the samples reduces the effect of radiation (at *p* < 0.05) in comparison to the control sample.

## 3. Discussion

It is well known that mammalian erythrocytes constitute an ideal cellular model for studying free radical lesions [29] since they are particularly sensitive to oxidative damage [30,31,32]. This is due to the presence of polyunsaturated fatty acids in their cell membranes and high concentrations of oxygen, as well as haemoglobin (Hb) [33]. Here, we studied the radiation-induced damage of erythrocytes and found a protective effect of C_60_(OH)_36_ enforcing the antioxidant defence system of RBC.

**Figure 4:**
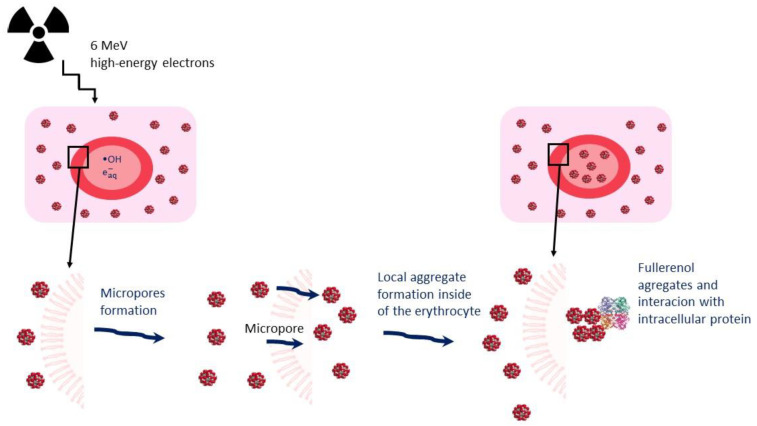
Proposed mechanism of micropore formation by IR and local aggregate formation of C_60_(OH)_36_ molecules and interaction with internal proteins inside erythrocyte.

Water radiolysis products generated by low linear energy transfer (LET) radiation cause 80–90% of cellular damage [34]; therefore, when cells and tissues are irradiated under aerobic conditions the hydroxyl radical (main product of water radiolysis) plays a major role in initiating cell damage, triggering primary and secondary radical reactions [35,36]. High-energy *γ*- and electron radiation can induce micropore formation [11]. These radiation-induced pores are usually smaller than the size of a haemoglobin molecule but large enough to allow for the passage of inorganic ions [27] and, plausibly, C_60_(OH)_36_ molecules (or ions). This process may result from lipid peroxidation or denaturation and the aggregation of integral proteins [37]. We suggest that the formation of micropores induced by IR could facilitate the penetration of C_60_(OH)_36_ molecules into the erythrocyte, enabling the local aggregate formation and, consequently, leading to the modulation of the activity of antioxidant enzymes, as shown in Figure 4.

Earlier findings from other laboratories suggest that polyhydroxylated fullerenes can scavenge ROS and stimulate the antioxidant system, making them promising compounds to be used as radioprotecting agents [7,14,38]. Very recently we have reported on the stimulating effect of C_60_(OH)_36_ on the antioxidant system inside human erythrocytes [9]. The mechanism of the radioprotective action of fullerenols proposed by Djordjevic and Bogdanovic [39] relies not only on the ability to scavenge RFTs, but also on stimulating antioxidant enzymes’ activity. Bogdanovic et al. [21] and Cai et al. [7] documented that ionising radiation causes a decrease in GSH concentration, superoxide dismutase (SOD) activity and GPx activity. They found that the administration of fullerenol before irradiation prevented a decrease in the activity of these enzymes, and our present results confirm this observation.

In the present study, the activity of selected antioxidant enzymes was monitored in erythrocytes incubated with C_60_(OH)_36_ and irradiated with doses of 650 and 1300 Gy. Radiation resulted in an increased concentration of total -SH groups measured as Parameter C accordingly by 27% and 38% for doses 650 Gy and 1300 Gy (Figure 1A). This probably caused a decrease in GPx activity by 44% (Figure 2A) due to its damage and increased CAT activity by 13%, only when samples were irradiated with 650 Gy (Figure 1B). The decomposition of H_2_O_2_ may occur not only through the enzymatic activity of CAT, but also by the action of haemoglobin, which, apart from playing a role as an electron carrier in biological systems has been shown to possess enzyme-like catalytic activity [40,41]. Zhao et al. evidenced that the adsorbed haemoglobin could transfer electrons directly to the carbon nanotubes interface. The rate constant for such heterogeneous electron transfer is 0.062 s^−1^ [42]. In our experiments, C_60_(OH)_36_ bound to irradiated haemoglobin might modify its structure or might be a linker facilitating the electron transfer between H_2_O_2_ and haemoglobin, contributing to its increased catalytic activity, and resulting in an accelerated decomposition of H_2_O_2_, as can be seen in Figure 1B.

The results presented in Figure 2A suggest that C_60_(OH)_36_ protects GPx from the damage induced by ROS generated during irradiation. Another reason for the maintained GPx activity may be the protective effect of C_60_(OH)_36_ on the cell membrane, as we proposed earlier [9]. 

NADPH is an important reductive equivalent protecting CAT from inactivation and used as a cofactor by GSR [9]. Some useful information can be gained from the experiments with erythrocytes incubated in a glucose-free medium, causing the inhibition of the pentose phosphate pathway, and responsible for most of the NADPH production necessary for the proper functioning of antioxidant enzymes [9]. In the above system, the dose-dependent decrease in GSH level (Figure 1A), as well as the consumption of NADPH by catalase, resulted in a decrease in GSR activity, while the presence of C_60_(OH)_36_ protected GSR from radiation-induced damage, as can be seen in Figure 2B. The ability of C_60_(OH)_36_ to protect CAT from IR-induced damage is presented in Figure 1B. Presumably, this protective and stimulatory effect can be assigned to the direct interaction of the nanoparticles with studied proteins, or because of the transfer of hydrogen atoms by C_60_(OH)_36_ molecules to the NADP^+^ molecule. This process could allow the maintenance of the appropriate level of NADPH. 

Ionizing radiation causes cellular dysfunction that affects the two main membrane components in erythrocytes: lipids and proteins [43]. This causes changes in membrane permeability not only to electrolytes, but also to non-electrolytes, resulting in haemolysis [44,45]. Changes in the microviscosity after the irradiation are manifested as a decrease in microviscosity by about 9% for the dose of 650 Gy and by about 17% for the dose of 1300 Gy, see Table 1. The reduced microviscosity may be a consequence of damage to the cytoskeleton proteins and/or the induced formation of micropores in the membrane enabling the efflux of electrolytes [45]. The results of measurements for samples incubated with fullerenol and then irradiated clearly demonstrate the protecting effect of C_60_(OH)_36_. This observation well corroborates the radioprotective action of C_60_(OH)_36_ towards cellular membranes evidenced previously by us and explained as due to the relatively strong adsorption of fullerenol to the components of membranes [11]. Thus, C_60_(OH)_36_ plausibly decreases the possibility of the formation of micropore and protects against negative changes in microviscosity.

Glutathione transferase is sensitive to oxidative stress [46] that is manifested as increased activity induced by ROS, and our results (experiments without fullerenol) indicate that IR causes a non-significant increase in GST activity after 2 h of irradiation. Previous studies by Doukali et al. [47] revealed that medical staff exposed to a low IR level were at risk of significant oxidative stress that was enhanced by GSTs polymorphisms in their erythrocytes. On the other hand, Cholon et al. [48] found that the ionizing radiation decreased the amount of cytosolic pi family member of GST without affecting alpha GST levels. The data suggested that GST cytosolic family members are involved in the cellular response against oxidative stress generated by ionizing radiation. We evidenced that C_60_(OH)_36_ significantly decreases the GST levels within the first 2 h after IR treatment in comparison to the samples irradiated without C_60_(OH)_36_. This effect could be assigned to the binding of fullerenol to cysteine residues, resulting in the modification of the cysteine residue at position 47, which is located near the active centre and caused a decrease in GST activity [48,49,50]. In our previous study, we also evidenced that C_60_(OH)_36_ itself caused a significant reduction in GST activity [5]. The most probable explanation of these results could be the interaction of C_60_(OH)_36_ with the active site of the enzyme.

## 4. Materials and Methods

### 4.1. Chemicals

The synthesis of C_60_(OH)_36_ by the organic solvent-free method from C_60_, NaOH, and 30% H_2_O_2_, as well as its purification and analysis of the product are described in [11]. The characteristics of the fullerenol (infrared, NMR including MALDI-TOF measurements, DLS and AMF), as well as the methodology of purifying fullerenol from sodium ions is described in our previous articles [11]. The size of C_60_(OH)_36_ measured by DLS indicates a peak at ca. 40 nm, and measurements by AFM (fullerenol dried on mica) show an average diameter of 2–10 nm. The difference between DLS and AFM, as well as the size distribution and long-term stability of obtained C_60_(OH)_36_ in water, are discussed in our previous publications [11,51].

Amberlite MB20 was purchased from Sigma-Aldrich (St. Louis, MO, USA). The highest purity reagents available were used in all the experiments. Inorganic salts, acids and bases were purchased from Avantor Performance Materials Poland (Gliwice, Poland) or Chempur (Piekary Śląskie, Poland). Buffers and aqueous solutions used to perform the experiments described in this paper were obtained with Mili-Q water (Mili-Q Integral water station, Milipore, Billerica, MA, USA).

### 4.2. Irradiation Conditions

Human erythrocyte suspensions (Ht = 2%) in PBS (pH 7.4) were exposed under air to high-energy electrons from the 6 MeV ELU-6 linear accelerator. Pulse radiolysis was performed with 17 ns electron pulses, which delivered doses of 65 Gy each. The total doses absorbed were 0.65 kGy or 1.3 kGy, as evaluated using a Rogowski coil. The measurements were performed 2 h after the irradiation.

### 4.3. Sample Preparation

Erythrocytes were isolated from the leukocyte-platelet coagulum by differential centrifugation (4 °C, 5 min, 3500 rpm). Blood was collected from healthy donors from the Regional Centre for Blood Donation and Haematology in Lodz. The obtained erythrocytes were washed three times with cooled 145 mM NaCl + 10 mM phosphosodium (PBS pH 7.4). Then, the haematocrit was measured by the microcapillary method, and the 2% haematocrit was used for further determinations.

The hemolysate was prepared by adding distilled water. To determine the activity of CAT: 0.5 mL of erythrocytes +1 mL of distilled water were mixed, incubated at −20 °C −30 min, and centrifuged −5 min, 7000 rpm). To determine the activity of SOD, GPx, GR and GST: 0.5 mL of the erythrocyte suspension was centrifuged (5 min, 7000 rpm), 0.48 mL of the solution +0.5 mL of water was taken, mixed, and incubated at 20 °C −15 min, incubated at 25 °C −10 min, and centrifuged (5 min, 7000 rpm). Hemolysates were stored at −70 °C.

The haemoglobin concentration was determined using the Drabkin method in 96-well plates. A total of 280 µL of 0.03% K_3_Fe(CN)_6_, 0.1% NaHCO_3_, 0.005% KCN (Drabkin’s reagent) was added to 20 µL of haemolysate (study sample) or 20 µL of distilled water (blank). The absorbance at λ = 541 nm was measured.

The test samples were prepared by adding the C_60_(OH)_36_ solution (starting concentration 5 mg/mL in PBS) to a 2% erythrocyte suspension to obtain a final concentration: 150 µg/mL was added to the erythrocyte suspension 1 h before the irradiation.

### 4.4. CAT Activity Determination

A total of 3 mL of 0.018 M hydrogen peroxide in 0.05 M phosphate buffer (pH 7.0) was added to the spectrophotometric cuvette to obtain an absorbance (λ = 240 nm) of about 1. To determine the CAT activity, 15 µL of hemolysate was added and the depletion of absorbance was measured (λ = 240 nm) within 1 min. CAT activity was expressed as mg of haemoglobin.

### 4.5. Glutathione Peroxidase Activity Determination

An indirect method was used to determine GPx. 250 µL of phosphate buffer equilibrated in 37 °C (0.1 M pH 7.0 with 0.1 mM EDTA) was mixed with 50 µL of 10 mM GSH, 50 µL of glutathione reductase (2.4 units/mL), 50 µL of 1, 5 mM NADPH in 0.1% NaHCO_3_ solution, and 50 µL of hemolysate (with the addition of 2 µL of 100 mM sodium azide to inhibit catalase and 2 µL of a mixture of 60 mM K_3_Fe(CN)_6_ and 48 mM solutions of KCN converting haemoglobin to cyanomethemoglobin). Then, 50 µL of 12 mM t-butyl hydroperoxide was added to initiate the reaction and the depletion of absorbance was measured (λ = 340 nm) within 3 min. The GPx activity was expressed as mg of haemoglobin.

### 4.6. Determination of Glutathione Transferase Activity

After incubating the RBC suspension with C_60_(OH)_36_ and irradiation (650 and 1300 Gy), the erythrocytes were subjected to haemolysis, and GST activity was measured. A mixture of 50 µL of 0.02 GSH solution, 50 µL of CDNB solution (0.02 M solution of 1-chloro-2,4-dinitrobenzene was dissolved in ethanol) and 50 µL of hemolysate was used. The increase in the depletion of absorbance was measured (λ = 340 nm) within 3 min. The glutathione transferase activity was expressed as mg of haemoglobin.

### 4.7. Determination of Glutathione Reductase Activity

After incubating the RBC suspension with C_60_(OH)_36_ subjected to irradiation, the erythrocytes were haemolysed and GSR activity was measured. To 1000 µL of 2.2 mmol/L oxidized glutathione solution was added 40 µL of hemolysate. After thorough mixing, 200 µL 0.17 mmol/L NADPH was added. The depletion of absorbance was measured (λ = 340 nm) within 3 min. GPx activity was expressed as mg of haemoglobin.

### 4.8. Erythrocyte Microviscosity Identification

**Figure 5:**
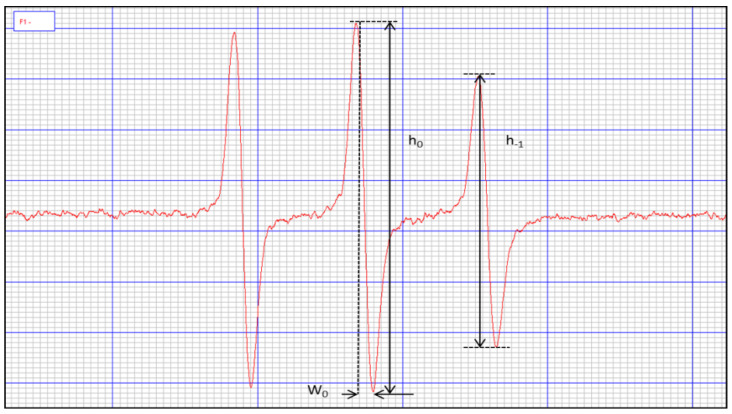
View of Tempamine marker.

For the determination of erythrocyte microviscosity, a 50% haematocrit was used, prepared by centrifugation of 1.6 mL of the incubated erythrocyte suspension and subsequent removal of 1.54 mL of the supernatant. To the remaining 60 µL, 0.6 µL of spin marker 10 mM was added and shaken for 30 min at room temperature. The samples were then washed with 1 mL of 80 mM potassium ferricyanide, centrifuged, and the EPR spectra of the samples, as well as the spectrum of the tracer in PBS, were determined, see Figure 5. The rotational correlation time of the tracer was calculated from the formula:

(1) $$\tau_{R}=kW_{0}\left(\sqrt{\frac{h_{0}}{h_{-1}}}-1\right)$$

where: *k* = 6.5 × 10^−9^ s/mT;

*W*_0_ =width of the spectral centre line;

*h*_0_ = height of the spectral mid-field line;

*h*_−1_ = height of the high-field line of the spectrum.

The viscosity of the erythrocyte interior was calculated from the following equation:

(2) $$\frac{\tau_{R}}{\tau_{B}}=\frac{\eta_{R}}{\eta_{B}}$$

where: *τ_R_*—rotational correlation time of the tracer inside erythrocytes; *τ_B_*—rotational correlation time of the tracer dissolved in a solution of known viscosity; *η_R_*—erythrocyte inner viscosity; and *η_B_* is the viscosity of the known solution.

### 4.9. Total Concentration of -SH Groups in Erythrocytes

Incubated erythrocytes with 2% haematocrit were diluted to a haematocrit of 0.75% with a final volume of 160 µL. Then, 0.45 µL of tracer RSSR spin tracer was added so that the final concentration in the sample was 80 µmol. After 3 min of incubation with the tag, EPR spectra were obtained similarly, as described previously [11].

## 5. Conclusions

Our results indicate that fullerenols used under certain conditions may affect the antioxidant proteins in human erythrocytes as well as their microviscosity when subjected to high-energy electrons evoked by IR. These findings support the notion of Tian et al. [52], suggesting that nanoparticles, such as fullerenols, act on erythrocyte membranes not only through the attachment to the membrane surface, but also via insertion into the membrane or entrance into the cell according to the different chemical properties, thus, affecting the protein content and conformation, resulting in further change in the morphology, structure, and function of erythrocytes. Overall, we documented the radioprotective effect of C_60_(OH)_36_, which is expressed as the protection and stimulation of the antioxidant enzymatic system against the IR-induced oxidative damage of human erythrocytes. As an outlook, a recommendation can be made for more detailed studies on the effect of selected water-soluble derivatives of fullerenes on human tissues and organs, as well in animal models to confirm their radioprotective potential. Hence, we demonstrated a high potential for the radioprotective studies based on C_60_(OH)_36_ application, which indicates the promising prospect of using highly hydroxylated fullerenes in modern radiotherapy.

## Figures and Tables

**Table 1:** η values determined with Tempamine use in a suspension of erythrocytes irradiated with 650 or 1300 Gy, incubated before irradiation with fullerenol at 150 µg/mL for 1 h at 37 °C and then irradiated with 650 and 1300 Gy. Measurements were taken 2 h after the irradiation.

η		SD
[Pa·s]		
2 h after the irradiation		
Control	4.72	±0.22
0.65 kGy	4.31	±0.29
0.65 kGy + C_60_(OH)_36_	4.62	±0.18
1.3 kGy	3.91 *	±0.22
1.3 kGy + C_60_(OH)_36_	4.42	±0.13

* Statistically significant values in relation to control sample at *p* < 0.05.

## Data Availability

Not applicable.
